# Germ Cell Responses to Stress: The Role of RNP Granules

**DOI:** 10.3389/fcell.2019.00220

**Published:** 2019-10-01

**Authors:** Jennifer A. Schisa

**Affiliations:** Department of Biology, Central Michigan University, Mount Pleasant, MI, United States

**Keywords:** stress, germ line, RNP granules, phase transition, oocytes, sperm, stress granule, P-body

## Abstract

The ability to respond to stress is critical to survival for animals. While stress responses have been studied at both organismal and cellular levels, less attention has been given to the effect of stress on the germ line. Effective germ line adaptations to stress are essential to the propagation of a species. Recent studies suggest that germ cells share some cellular responses to stress with somatic cells, including the assembly of RNP granules, but may also have unique requirements. One fundamental difference between oocytes and sperm, as well as most somatic cells, is the long lifespan of oocytes. Since women are born with all of their eggs, oocytes must maintain their cellular quality over decades prior to fertilization. This prolonged meiotic arrest is one type of stress that eventually contributes to decreased fertility in older women. Germ cell responses to nutritional stress and heat stress have also been well-characterized using model systems. Here we review our current understanding of how germ cells respond to stress, with an emphasis on the dynamic assembly of RNP granules that may be adaptive. We compare and contrast stress responses of male gametes with those of female gametes, and discuss how the dynamic cellular remodeling of the germ line can impact the regulation of gene expression. We also discuss the implications of recent *in vitro* studies of ribonucleoprotein granule assembly on our understanding of germ line responses to stress, and the gaps that remain in our understanding of the function of RNP granules during stress.

## Introduction

The ability to respond to stress is essential for the survival of a species. While stress responses have long been studied at a physiological level, major inroads into understanding cellular responses to stress have occurred more recently. An important adaptive response is the reprograming of gene expression, and one conserved feature of cellular stress response is the assembly of stress-induced RNP (ribonucleoprotein) granules, including an increase in the formation of *de novo* Processing-bodies (P-bodies) and the assembly of stress granules (Teixeira et al., 2005; Huch and Nissan, 2017; Ivanov et al., 2019). Stress granules are complexes of mRNA and RNA-binding proteins that assemble in response to environmental stresses including oxidative stress, heat stress, and osmotic stress (Kedersha and Anderson, 2002). They can also be distinguished from other RNP granules, as they are stalled translation initiation complexes regulated by the phosphorylation of eIF2alpha, and they have been most well characterized in yeast and mammalian cell culture. Stress induces disassembly of the polysome, and the released mRNPs are sequestered in stress granules to protect mRNA from degradation and prevent translation until the stress subsides (Kedersha and Anderson, 2002). A recent study examining single mRNAs during arsenite stress shows that mRNAs stably associated with stress granules are non-translating (Moon et al., 2018). Stress granules have also been implicated in protein quality control mechanisms, with deregulation contributing to neurodegenerative diseases, among other disorders (Alberti et al., 2017). P-bodies share some components and properties with stress granules, but are distinct in composition, behavior, and proposed functions (Ivanov et al., 2019). P-bodies were initially identified as having roles in RNA decay (Sheth and Parker, 2003); however, current studies indicate P-bodies may have a larger role in housing transiently repressed mRNAs during stress (Hubstenberger et al., 2017).

The germ line has distinct functions and properties from the soma, most notably the need to accurately transmit genetic information between generations and the requirement of pluripotency. Given its essential role in the survival of a population, it is not surprising that the germ line has evolved cellular adaptations to stress. The critical nature of stress responses applies not only to the survival of gametes, but also to the maintenance of cell quality that can be compromised, leading to serious birth disorders or diseases appearing during adulthood (Latham, 2016). The germ line may have unique requirements in regulating gene expression, as large pools of maternal mRNAs accumulate in oocytes, many of which remain untranslated until post-fertilization. Thus, some germ line stress responses may be unique to germ cells, and some appear to be specific to oocytes. In metazoans, sperm are generally produced by uninterrupted meiosis of spermatids. In contrast, oocytes arrest in meiosis for extended periods of time prior to fertilization; this arrest varies from days in *Drosophila*, to years in *Xenopus*, to decades in humans (Masui and Clarke, 1979). Extended meiotic arrest can eventually lead to infertility and a host of birth defects that are correlated with increased maternal age (Hunt and Hassold, 2010). Oocytes are also sensitive to temperature, osmolarity, oxygen, nutrient restriction, and pH. Variations in these factors in mammals can trigger endoplasmic reticulum (ER) stress, the unfolded protein response (UPR), and epigenetic changes (Latham, 2016). One cellular stress response that can be protective for gametes is the assembly of stress-induced RNP granules. Stress-induced RNP granules have been described in vertebrate and invertebrate, and male and female, germ lines; however, their function remains largely unknown. One interesting possibility is that germ line RNP granules may prevent somatic development, as occurs with germ granule components during normal development in *Caenorhabditis elegans* (Updike et al., 2014). Gaining a better understanding of how the male and female germ lines respond to stress, and the regulation and function of RNP granules in particular, should ultimately improve our understanding of human fertility problems, and may also contribute to insights of related RNP complexes in all cell types.

## Response to Extended Meiotic Arrest in Eggs

One type of stress unique to oocytes is extended meiotic arrest, as described above. Female mammals are born with all of their oocytes arrested in meiosis I, and the effects of increased maternal age, or more correctly egg-age, are broadly understood to be correlated with increased risk, e.g., higher rates of non-disjunction resulting in aneuploidy (Hunt and Hassold, 2008; Franasiak et al., 2014; Cimadomo et al., 2018). Several excellent reviews discuss the impact of maternal age on oocyte quality, focusing on mitochondrial dysfunction, chromosomal abnormalities, and reduced ovarian reserve (Heffner, 2004; Franasiak et al., 2014; Cimadomo et al., 2018). More recent findings indicate that in some organisms the response to extended meiotic arrest shares commonalities with responses to environmental stresses, including the assembly of RNP granules.

**FIGURE 1:**
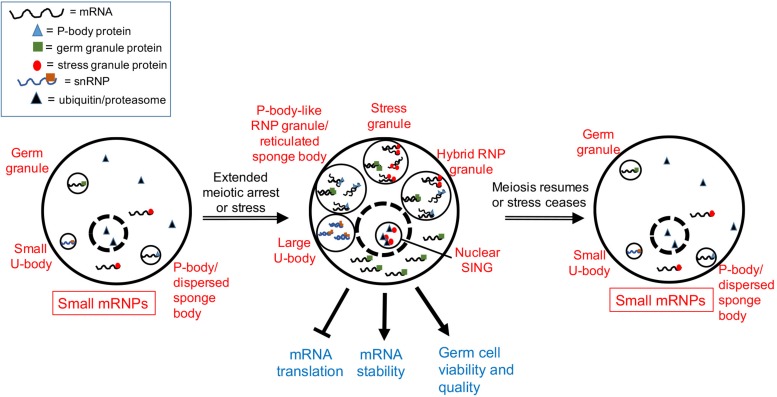
Model showing a composite of RNP granule stress responses during gametogenesis. Depending on the organism and the type of stress (see text), one or more of the five responses can occur in germ cells. Dashed circle is the nucleus. Stress granules assemble in heat-stressed mouse spermatocytes. Hybrid RNP granules form in the *Caenorhabditis elegans* germ line and contain germ granule proteins, as well as P-body and stress granule proteins. P-body-like RNP granules, similar to reticulated sponge bodies, assemble in *Drosophila* egg chambers and lack stress granule markers. Large U bodies associate with P-bodies in stressed *Drosophila* oocytes. Nuclear SINGS are stress-induced nuclear granules in the *C. elegans* germ line that may represent localized protein degradation.

In young adult *C. elegans* hermaphrodites, oocytes undergo meiotic maturation, and ovulation every 23 min. However, after 3–4 days of adulthood the sperm supply becomes depleted, resulting in oocytes in an arrested state of meiosis I that accumulate in the gonad arms (McCarter et al., 1999). The distribution of many maternal mRNAs and RNA-binding proteins changes in the arrested oocytes, with the assembly of large complexes of P-body-like RNP granules (Schisa et al., 2001; Jud et al., 2008; Noble et al., 2008; Figure 1). The assembly of RNP granules in oocytes is evolutionarily conserved in several gonochoristic species in the *Caenorhabditis* clade, consistent with a role in protecting maternal mRNAs during extended meiotic arrest prior to females finding males (Jud et al., 2007). Components of the RNP granules include P-body proteins, stress granule proteins, germ granule proteins, other RNA-binding proteins, and maternal mRNAs; thus, they can be considered hybrid RNP granules (Schisa, 2014; Figure 1). The RNP granules disassemble in response to a new supply of sperm to the worm that triggers the resumption of meiosis, and greater than 90% of arrested oocytes that contain RNP granules can eventually give rise to viable embryos (Andux and Ellis, 2008; Jud et al., 2008). Based on their composition, the hypothesis for RNP granule function is to maintain maternal mRNAs and oocyte quality during delays in fertilization (Figure 1). In support of this hypothesis, the disruption of RNP granule assembly correlates with modest, but significant, decreases in oocyte quality (Wood et al., 2016).

Similar cellular responses appear to occur in the *Drosophila* germ line as well. Sponge bodies are cytoplasmic structures in *Drosophila* nurse cells and oocytes that have ER-like vesicles and are implicated in post-transcriptional control of gene expression (Wilsch-Brauninger et al., 1997; Snee and Macdonald, 2009). Sponge bodies are dispersed in 3–4 day-old females, but in virgin females, the sponge bodies in the germ line become reticulated structures. In dispersed sponge bodies some RNA-binding proteins like Orb are dispersed throughout the cytoplasm, and other P-body markers such as the de-capping activator/RNA helicase Me31B/RCK are enriched in small puncta. In contrast, in reticulated sponge bodies, much larger bodies of Me31B and Orb aggregate, with lower levels of these proteins detected in the cytoplasm (Wilsch-Brauninger et al., 1997; Lue et al., 1999; Snee and Macdonald, 2009). Based on an absence of the stress granule marker poly (A) binding protein, and the presence of Me31B and Dcp1, the reticulated sponge bodies are considered to be more similar to P-bodies than stress granules (Figure 1). That the composition of sponge bodies includes many post-transcriptional regulatory factors, has led to a hypothesis that they store RNPs or contribute to trafficking of RNPs. The remodeling of RNP complexes during extended meiotic arrest in worms and flies suggests a requirement for additional post-transcriptional gene regulation. It will be intriguing to determine if similar RNP granules assemble during extended meiotic arrest in mammalian oocytes and how they function.

## Responses to Environmental Stresses in Male and Female Germ Lines

### Heat Stress

The germ line must be able to respond to a variety of environmental stresses in order to generate viable offspring. Several adaptive responses to heat stress have been documented in both male and female germ lines, and in vertebrates and invertebrates. In mammals, the testis is normally located outside the body and is therefore at a lower temperature than the core body; exposing the testis to heat stress can ultimately lead to DNA fragmentation and apoptosis of germ cells (Lue et al., 1999; Rockett et al., 2001; Kim J.H. et al., 2013; Fan et al., 2017). One protective response to heat stress in mice is the phosphorylation of eIF2α, a subunit of eukaryotic initiation factor 2, which leads to a general halt of translation with the exception of select mRNAs, and the assembly of DAZL-containing stress granules in spermatogonia and early spermatocytes (Krishnamoorthy et al., 2001; Kim B. et al., 2012, 2013; Figure 1). DAZL is a conserved germ cell-specific translational regulator and is hypothesized to protect against heat stress by regulating mRNA transport or translational regulation (Collier et al., 2005; Kim B. et al., 2012). In the absence of DAZL and stress granule formation, the percent of apoptotic cells in testes increases significantly, suggesting DAZL-containing stress granules protect germ cells (Kim B. et al., 2012). In the invertebrates *C. elegans* and *Drosophila*, heat stress also has severe impacts on sperm function and viability (Parsons, 1973; Chakir et al., 2002; Petrella, 2014; Gouvea et al., 2015). Interestingly, male worms cannot recover their fertility after being downshifted to a moderate temperature which is in contrast to *Drosophila* and mammals. Assembly of stress-induced RNP granules has not been described in invertebrate male gametes to date; thus, alternative protective mechanisms may contribute the functions performed by DAZL-granules in mice.

The female germ cells of *Drosophila* and *C. elegans* are also sensitive to heat stress, displaying decreases in proliferation and increases in apoptosis (Gruntenko et al., 2003; Salinas et al., 2006; Petrella, 2014). One potentially protective response is the assembly of RNP granules in *Drosophila* egg chambers and in *C. elegans* germ lines. In the *Drosophila* ovary, heat stress induces granules of Hsp70 that colocalize with the RNA-binding protein Rbfox1/A2bp1, suggesting Rbfox1 can localize to stress granules under certain stress conditions (Kucherenko and Shcherbata, 2018; Figure 1). Heat stress also induces large-sized U bodies that associate with P-bodies (Buckingham and Liu, 2011; Figure 1). U bodies are cytoplasmic structures that are sites of snRNP assembly that associate with P-bodies (Liu and Gall, 2007; Cauchi, 2011). The increased size is speculated to allow for more effective storage of snRNPs during stress.

Heat stress induces granules in the oocytes and distal germ line of *C. elegans* that are similar, but not identical, to the hybrid RNP granules induced by extended meiotic arrest (Jud et al., 2008). Components include markers of stress granules: PAB-1 (poly A binding protein), TIAR-2 (formerly named TIA-1, T-cell-restricted intracellular antigen protein), and TIAR-1 (Jud et al., 2008; Huelgas-Morales et al., 2016), and cycloheximide treatment during heat stress reduces the abundance of TIAR-1 granules, similar to its effects on stress granules (Huelgas-Morales et al., 2016). Components of the granules also include markers of P-bodies: DCAP-2/Dcp1, CAR-1/RAP55, and CGH-1/RCK, other RNA-binding proteins such as MEX-3, and the germ granule protein VBH-1 (Jud et al., 2008; Paz-Gomez et al., 2014). Thus, it is not clear if the stress-induced granules are bona fide stress granules. Interestingly, the germ granule proteins PGL-1 and GLH-1 react oppositely to heat stress, and their distribution becomes diffuse throughout the cytoplasm of oocytes (Jud et al., 2008; Figure 1). Similarly in embryos, the PGL-1 phase of germ granules, but not the scaffolding protein MEG-3, dissolves at elevated temperatures (Smith et al., 2016). Thus, some of the RNA-binding proteins in meiotic arrest-induced granules have different properties at high temperature, perhaps reflecting a less-liquid granule core surrounded by a more soluble outer shell structure, similar to what has been observed in yeast and mammalian stress granules in culture (Jain et al., 2016). The function of heat stress-induced RNP granules may be protective but remains unclear. In animals pre-treated by growth at moderately high temperatures prior to heat shock, TIAR-1 granules assemble normally in oocytes but fail to assemble in the gonad core. Despite this failure, germ cells survive heat shock well, suggesting the gonad core granules are independent of the ability of TIAR-1 to protect germ cells (Huelgas-Morales et al., 2016). This result may also suggest that the RNP granules associated with pachytene-stage cells in the worm gonad core function differently or redundantly with those in maturing oocytes.

The deleterious effects of heat stress in the mammalian female germ line have been well characterized in *in vitro* fertilization livestock studies due to their economic significance (Hansen, 2009). In one study, exposure of bovine cumulus oocyte complexes to elevated temperature resulted in a decreased likelihood of oocytes developing to the blastocyst stage (Edwards and Hansen, 1996). Subsequent studies have extended this observation, demonstrating negative impacts of heat stress on oocyte development for cattle, pigs, water buffalo, rabbits, and camels (Waiz et al., 2016; Garcia and Argente, 2017; Hale et al., 2017; Roth, 2017; Saadeldin et al., 2018). No studies to date have detected heat stress-induced RNP granule assembly in oocytes of vertebrate model systems. However, in zebrafish, the P54 RNA helicase, a marker of P-bodies in worms (CGH-1) and flies (Me31B), localizes to stress-induced granules in early embryos in response to heat stress. It was not determined whether the P54 granules assemble in the zebrafish primordial germ cells, and adult germ lines were not examined (Zampedri et al., 2016). If RNP granules have a critical function in the germ line during heat stress, one intriguing question for the future is to determine the extent to which the remodeling is conserved, and how this function is accomplished in germ lines lacking this stress response.

### Nutritional Stress

Nutritional stress is well-known to affect the development and quality of male and female gametes. Deficiencies in vitamin D, vitamin A, or zinc can lead to decreases in male fertility or increased apoptosis in the testes of mammals (Croxford et al., 2011; Boucheron-Houston et al., 2013; Sun et al., 2015; Fan et al., 2017). Protein starvation in *Drosophila* elicits a temporary reduction in proliferation rates during spermatogenesis, that interestingly recovers with prolonged protein starvation (McLeod and White, 2010; Yang and Yamashita, 2015). Positive effects of nutritional stress are seen in *C. elegans*, where short-term starvation in young male worms prevents age-related declines in sperm-production in progeny (Chou et al., 2019). This result demonstrates an intriguing transgenerational, and positive effect of nutritional stress, and it will be interesting to determine the extent to which changes in gene expression are involved.

Nutrition restriction induces several stress responses in invertebrate oocytes. When female *Drosophila* are starved of yeast, the rate of cell division in the germ line decreases, and the rate of cell death increases (Drummond-Barbosa and Spradling, 2001). Yeast starvation also induces dramatic changes in the size and distribution of sponge bodies. When mated flies are fed standard growth media supplemented with dried yeast, sponge bodies are dispersed in egg chambers as small puncta that appear similar to P-bodies; however, if the dried yeast is not provided, much larger, reticulated sponge bodies assemble (Snee and Macdonald, 2009; Figure 1). In a related study, in flies fed a protein-poor diet, large RNPs were detected that have several, but not all, of the components of reticulated sponge bodies (Shimada et al., 2011). These RNP granules also contain P-body markers (dDcp1, Pacman/XRN1, eIF4E) but not stress granule markers (Staufen, phosphorylated eIF2α). At the same time, microtubules become cortically condensed in the fly egg chamber, and dynein activity is required for the nutritional stress-induced cellular remodeling (Burn et al., 2015). The inability of egg chambers to form large RNPs is correlated with reduced survival of progeny, suggesting the RNPs are protective (Burn et al., 2015). An independent study shows that P-bodies induced by starvation also contain the Rbfox1/A2bp1 protein colocalized with Pacman, and the assembly of the RNP granules along with *miR-980*-regulation of Rbfox1 levels is positively correlated with increased cell survival (Kucherenko and Shcherbata, 2018). Nutrition deprivation also induces large-sized U bodies in *Drosophila* oocytes that correlates with decreased splicing activity (Buckingham and Liu, 2011; Cauchi, 2011).

The germ line response to starvation in *C. elegans* oogenesis appears similar to that in *Drosophila*. Germ cell proliferation halts, and apoptosis is induced 2.5-fold via apparent changes in gene expression of the apoptotic machinery (Salinas et al., 2006; Lascarez-Lagunas et al., 2014). Starvation also induces the assembly of RNP granules in oocytes and the gonad core that are similar to those induced by heat stress (Huelgas-Morales et al., 2016), as well as nuclear granules in oocytes referred to as SINGS (stress induced nuclear granules) (Sampuda et al., 2017). The SINGS contain the stress granule marker TIAR-2, ubiquitin, and proteasome. Their appearance is correlated with protein misfolding, leading to a model that the SINGs may function as part of a protein quality control system. This report appears to be the first description of a stress-induced nuclear body in the germ line; however, heat stress induces the assembly of HSF-1 nuclear stress granules in worm somatic tissues (Morton and Lamitina, 2013).

Nutrition restriction in mammalian oocytes is one of several stresses that lead to ER stress signaling (ERSS) and the UPR (Latham, 2015). Adverse effects of ERSS and UPR on oocytes and embryos include changes in imprinting, gene expression, mitochondrial function, and in meiotic spindles. To combat the impact of the ER stress response a variety of ERSS inhibitors have been studied, with tauroursodeoxycholic acid (TUDCA) showing promising benefits (Abraham et al., 2012; Kim J.S. et al., 2012; Zhang et al., 2012). The use of resveratrol and other antioxidants *in vitro* or when injected into mothers can also improve the quality of oocytes in several mammalian species (Lee et al., 2010; Liu et al., 2013; Mukherjee et al., 2014).

In terms of RNP remodeling in the mammalian germ line, during mouse oocyte growth P bodies seem to disappear at the same time that subcortical aggregates (SCAs) assemble (Flemr et al., 2010). The SCAs are RNP complexes containing the RNA helicase DDX6/RCK and other RNA binding proteins; however, no studies to date have indicated that any type of stress impacts the assembly of SCAs or any other type of RNP granule.

## Conclusion and Future Prospects

The past few decades have been exciting in terms of identifying and characterizing a variety of RNP granules induced by stress. RNP granules have received additional attention as broadly conserved examples of phase separation. The preponderance of studies utilize yeast and mammalian cell culture for their ease; however, these studies are limited in the insights they can provide regarding the *in vivo* functions of RNP granules during stress. In particular, the germ line has unique requirements for regulating gene expression, and its adaptive stress responses may differ from those in somatic cells. At a basic level, we still do not know what the visible phase separation of RNP granules means at a molecular level. Many hypotheses exist as to whether stress-induced RNP granules assemble to: maintain the protein or RNA components within them, prime cells for rapid protein synthesis and recovery after stress ceases, reduce the concentration of RNA binding proteins in the bulk cytoplasm surrounding the granules, or whether the granules are just a consequence of more global remodeling occurring throughout the cell (Guzikowski et al., 2019). Creative *in vitro* studies have identified many types of interactions that promote phase separation outside of cells: protein-protein, RNA-RNA binding protein, roles of intrinsically disordered regions of proteins, RNA-RNA, and electrostatic interactions between RNA and protein. Triggers of phase separation *in vitro* also include the concentration of proteins and RNA, phosphorylation state, and salt concentration (Kato et al., 2012; Kwon et al., 2013; Su et al., 2016). Because RNP granules *in vivo* are more complex than those *in vitro*, the relative contributions of these various triggers are not yet clear. Complementary *in vivo* studies have also revealed regulators of RNP granule assembly. Chaperone proteins have now been implicated in regulating RNP granule assembly in both somatic cells and in the germ line (Spiess et al., 2004; Tam et al., 2006; Curran et al., 2009; Updike and Strome, 2009; Nadler-Holly et al., 2012; Hubstenberger et al., 2013; Jain et al., 2016; Wood et al., 2016). The role of single transcript RNPs in seeding stress-induced RNP granules is also worth exploring based on *Drosophila* studies showing germ granule assembly is regulated by recruitment of single transcript RNPs to homotypic clusters (Nielpielko et al., 2018). Autophagy has been implicated in the homeostasis of the chromatoid body, a unique RNP granule in mouse spermatocytes, and in regulating germ granule components in *C. elegans* (Da Ros et al., 2017; Min et al., 2019); therefore, it may also be worth investigating in the context of stress-induced RNP granules. Much remains to be discovered as to the regulation and function of RNP granules induced by stress in the germ line; however, the future is promising with advances in *in vitro* reconstitution assays, improved single molecule FISH methods, and increased attention on the impact of RNP remodeling on gene expression. Combinations of *in vitro* and *in vivo* approaches will likely be important to illuminate the function of stress-induced RNP granules in the germ line.

## Author Contributions

JS wrote the manuscript and prepared the figure.

## Conflict of Interest

The author declares that the research was conducted in the absence of any commercial or financial relationships that could be construed as a potential conflict of interest.
